# Incremental effect of coronary obstruction on myocardial microvascular dysfunction in type 2 diabetes mellitus patients evaluated by first-pass perfusion CMR study

**DOI:** 10.1186/s12933-023-01873-w

**Published:** 2023-06-28

**Authors:** Jin Wang, Zhi-Gang Yang, Ying-Kun Guo, Yu Jiang, Wei-Feng Yan, Wen-Lei Qian, Han Fang, Chen-Yan Min, Yuan Li

**Affiliations:** 1 Department of Radiology, West China Hospital, Sichuan University, 37# Guo Xue Xiang, Chengdu, 610041 Sichuan China; 2 Department of Radiology, Key Laboratory of Obstetric and Gynecologic and Pediatric Diseases and Birth Defects of Ministry of Education, West China Second University Hospital, Sichuan University, 20# Section 3, Renmin South Road, Chengdu, 610041 Sichuan China

**Keywords:** Type 2 diabetes mellitus, Obstructive coronary artery disease, First-pass perfusion, Microvascular dysfunction, Magnetic resonance imaging

## Abstract

**Background:**

Type 2 diabetes mellitus (T2DM) frequently coexists with obstructive coronary artery disease (OCAD), which are at increased risk for cardiovascular morbidity and mortality. This study aimed to investigate the impact of coronary obstruction on myocardial microcirculation function in T2DM patients, and explore independent predictors of reduced coronary microvascular perfusion.

**Methods:**

Cardiac magnetic resonance (CMR) scanning was performed on 297 T2DM patients {188 patients without OCAD [T2DM(OCAD −)] and 109 with [T2DM(OCAD +)]} and 89 control subjects. CMR-derived perfusion parameters, including upslope, max signal intensity (MaxSI), and time to maximum signal intensity (TTM) in global and segmental (basal, mid-ventricular, and apical slices) were measured and compared among observed groups. According to the median of Gensini score (64), T2DM(OCAD +) patients were subdivided into two groups. Univariable and multivariable linear regression analyses were performed to identify independent predictors of microcirculation dysfunction.

**Results:**

T2DM(OCAD −) patients, when compared to control subjects, had reduced upslope and prolonged TTM in global and all of three slices (all *P* < 0.05). T2DM(OCAD +) patients showed a significantly more severe impairment of microvascular perfusion than T2DM(OCAD −) patients and control subjects with a more marked decline upslope and prolongation TTM in global and three slices (all *P* < 0.05). From control subjects, through T2DM(OCAD +) patients with Gensini score ≤ 64, to those patients with Gensini score > 64 group, the upslope declined and TTM prolonged progressively in global and mid-ventricular slice (all *P* < 0.05). The presence of OCAD was independently correlated with reduced global upslope (β =  − 0.104, *P* < 0.05) and global TTM (β = 0.105, *P* < 0.05) in patients with T2DM. Among T2DM(OCAD +) patients, Gensini score was associated with prolonged global TTM (r = 0.34, *P* < 0.001).

**Conclusions:**

Coronary artery obstruction in the context of T2DM exacerbated myocardial microcirculation damage. The presence of OCAD and Gensini score were independent predictors of decreased microvascular function.

*Trial registration:* Retrospectively registered.

## Background

Type 2 diabetes mellitus (T2DM) is accompanied by multisystem micro- and macro-vascular complications [1, 2]. As one of the major risk factors for coronary artery, T2DM can affect coronary microvasculature as well as epicardial coronary arteries [3]. Growing evidence has underscored deleterious effects of coronary microvascular dysfunction (CMD) in T2DM patients, which might lead to adverse left ventricular (LV) remodeling, reduced myocardial function, and worse clinical outcomes [4, 5]. In addition, there is a markedly increased incidence of major adverse cardiovascular events (MACE) associated with coronary macrovascular complication, such as obstructive coronary artery disease (OCAD), in patients with T2DM [1, 2, 6]. Accumulating data have indicated that CMD is correlated with increased cardiovascular mortality and poor prognosis in T2DM patients with OCAD [7–9]. Thus, early identification of CMD among those patients might contribute to risk stratification and prognosis evaluation for optimal medical treatments [10, 11].

Several studies have demonstrated that microvascular resistance is influenced by the severity of epicardial artery stenosis [12–14]. Although structural, functional, and metabolism alterations of the microvasculature and extravascular changes might elucidate CMD [15–17], the pathophysiological mechanisms of CMD under the presence of coronary obstruction remain not fully understood. Since CMD is an independent predictor of MACE in T2DM patients [5], it is utmost significant to assess coronary microvascular function and understand the relationship between epicardial coronary obstruction and CMD among those patients.

First-pass cardiac magnetic resonance (CMR) perfusion has emerged as a sensitive, non-invasive and accurate imaging modality for monitoring myocardial microvascular function, which has been extensively validated in various diseases [18–20]. To the best of our knowledge, most studies have investigated effects of obesity or hypertension on myocardial perfusion in T2DM patients [21, 22], whereas the additive impact of epicardial coronary obstruction on myocardial microcirculation damage has rarely been studied in those patients [23]. Herein, we aimed to: 1) evaluate the impact of coronary obstruction on myocardial microcirculation function in T2DM patients, and 2) to investigate independent predictors of reduced coronary microvascular perfusion.

## Methods

### Study population

Patients with T2DM who underwent CMR examination at our institution were retrospectively enrolled in this study from January 2015 to April 2022. Invasive coronary angiography (ICA) examination was performed to diagnose OCAD. OCAD was defined on condition that angiographic evidence of ≥ 50% diameter stenosis showed in at least one major epicardial coronary artery [24]. According to the American Diabetes Association guidelines [25], T2DM was diagnosed and the diagnostic criteria were as follows: typical diabetes symptoms and random plasma glucose (PG) level ≥ 11.1 mmol/L, or fasting PG level ≥ 7.0 mmol/L, or 2-h PG level ≥ 11.1 mmol/L after a 75-g oral glucose tolerance test, or hemoglobin A_1c_ level ≥ 6.5 mmol/L. The Exclusion criteria included patients with type 1 diabetes mellitus, previous coronary artery bypass grafting or stenting, primary cardiomyopathy, valvular or congenital heart disease, severe renal dysfunction with an estimated glomerular filtration rate < 30 ml/min/1.73m^2^, any contraindication to CMR examination, and CMR image quality is unsuitable for diagnosis.

Ultimately, 297 T2DM patients (mean age, 58.95 ± 11.61 years; 197 male) were enrolled into the study and were classified into two groups: T2DM without OCAD [T2DM(OCAD −), n = 188] and T2DM with OCAD [T2DM(OCAD +), n = 109]. Age- and sex-matched individuals were enrolled served as the control group. The exclusion criteria of the control group were as follows: history of systematic or cardiovascular disease, known diabetes mellitus or impaired glucose tolerance, and abnormalities detected by CMR, such as abnormal ventricular motion, perfusion defect, and decreased LV ejection fraction (LVEF), etc. Finally, a total of 89 individuals (mean age, 56.38 ± 8.63 years; 51 male) were included in this study. This study protocol was approved by the Biomedical Research Ethics Committee of our hospital. Written informed consent was waived because of the retrospective nature of the study.

### Invasive coronary angiography

According to the standard Judkins technique, invasive coronary angiography was performed by experienced interventional cardiologists adopting radial or femoral artery approach. On the basis of the number of diseased coronary arteries with ≥ 50% stenosis, T2DM(OCAD +) patients were categorized as a one-, two-, or three-vessel disease. Coronary artery stenosis of ≥ 50% in left main coronary artery was regarded as three-vessel disease [26]. Based on the method described in the literature [27], Gensini coronary score was used for evaluating the severity of OCAD and was calculated by two independent experienced cardiologists.

### CMR protocol

CMR examination was performed on a 3.0 T whole-body scanner Trio Tim or MAGNETOM Skyra (Siemens Medical Solutions, Erlangen, Germany). All participants were examined in the supine position, and equipped with standard ECG-triggering device and 32-channel body phased array coils. During the end-inspiratory breath-holding period, continuous data acquisition was performed using a retrospective vector ECG gating technique. Cine imaging was performed in the short-axis slices, as well as the two-, three-, and four-chamber in the long-axis covering the whole LV from the base to the apex views using a balanced steady-state free precession (bSSFP) sequence. The following scanning parameters were used: repetition time [TR]: 2.8 ms or 3.4 ms, echo time [TE]: 1.2 ms, field of view [FOV]: 303 × 360mm^2^ or 284 × 340mm^2^, flip angle 50° or 38°, slice thickness 8 mm, and matrix size 162 × 192 or 174 × 208.

Subsequently, gadolinium-based contrast agent was intravenously injected at a dose of 0.2 mL/kg body weight (injection rate: 2.5 − 3.0 mL/s), then a 20 mL saline flush was injected immediately following contrast at a rate of 3.0 mL/s. Rest first-pass perfusion images in three standard short-axis slices (basal, mid-ventricular, and apical) and in one slice of four-chamber view were acquired using an inversion recovery prepared echo-planar imaging sequence. The following scanning parameters were used: repetition time [TR]:163.2 ms or 149.8 ms, echo time [TE]: 1.06 or 0.99 ms, field of view [FOV]: 240 mm × 320 mm^2^ or 270 × 360mm^2^, flip angle 10°, slice thickness 8 mm, and matrix size 132 × 176 or 144 × 192.

### CMR data analysis

CMR images analysis was evaluated offline using commercial software (cvi^42^, Circle Cardiovascular Imaging Inc., Calgary, Alberta, Canada) by two experienced radiologists. For each participant, LV end-diastolic volume (LVEDV), LV end-systolic volume (LVESV), LV stroke volume (LVSV), LV myocardial mass, and LVEF were calculated using the above-mentioned software by manually outlined the epicardial and endocardial borders of the LV myocardium on a stack of short-axis cine images at the end-systolic and end-diastolic phases. LV papillary muscles and moderate bands were excluded from LV myocardial mass and included in LV cavity. LV volumes and mass were corrected for body surface area (BSA), which was calculated using the Mosteller equation [28].

**Fig. 1:**
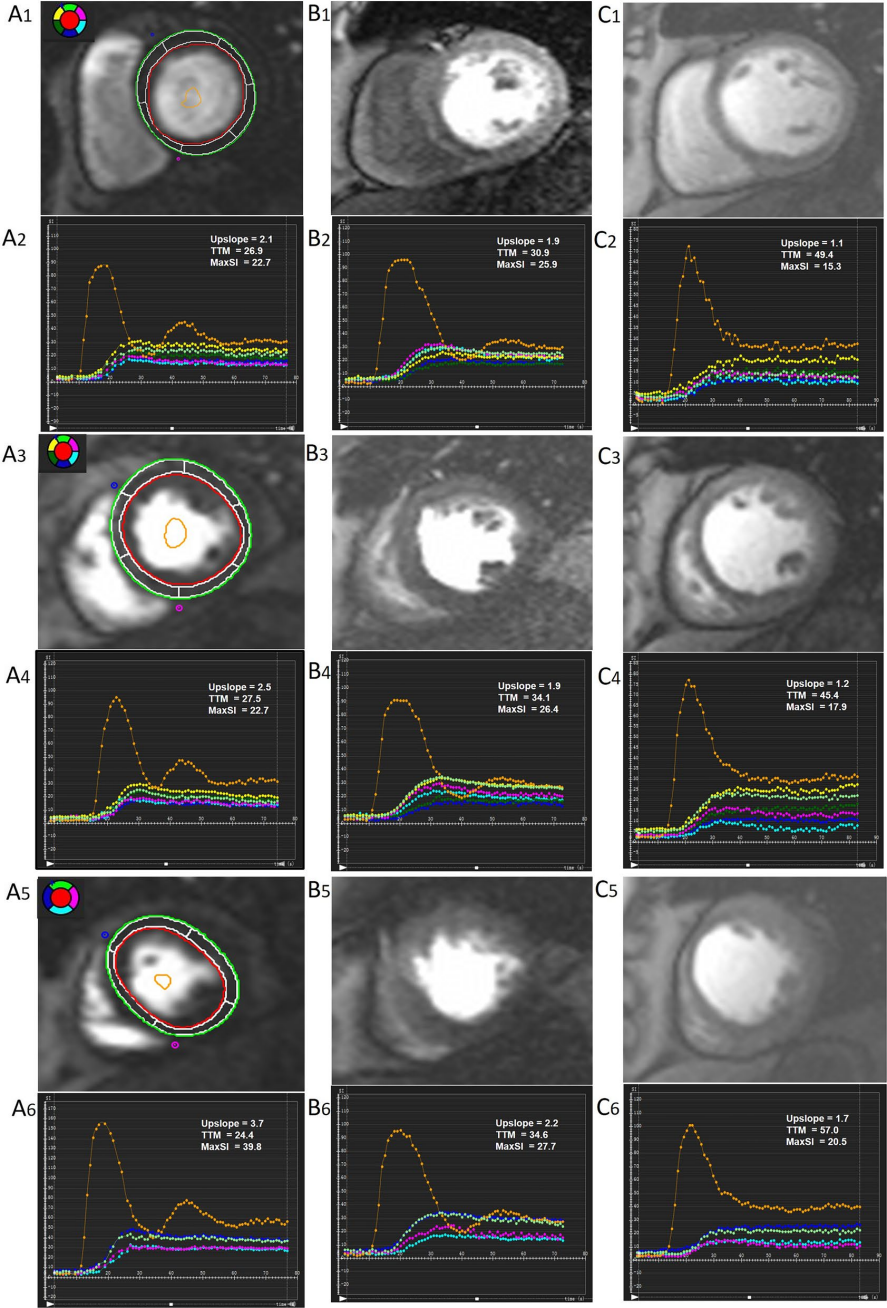
Representative first-pass perfusion CMR images and signal intensity-time curves in a control subject (**A1**–**A6**), T2DM(OCAD −) patient (**B1**–**B6**), and T2DM(OCAD +) patient (**C1**–**C6**). Signal intensity-time curves were acquired from basal, mid-ventricular, and apical slices. *T2DM* type 2 diabetes mellitus; *OCAD* obstructive coronary artery disease. *MaxSI* max signal intensity, *TTM* time to maximum signal intensity

For analyzing LV myocardial perfusion, signal intensity-time curves, including each myocardial segment based on the 16-segment model (Bull’s eye plot) and the blood pool, were generated by manually delineated epicardium, endocardium and blood pool counters in first-pass perfusion images of all three short-axis slices (the basal, middle, and apical) with exclusion of papillary muscles and moderator bands. Each myocardial segmental perfusion parameters, including upslope, time to maximum signal intensity (TTM) and maximum signal intensity (MaxSI), were consequently obtained from myocardial signal intensity-time curves (Fig. 1, A2-C2, A4-C4, A6-C6). All LV global myocardial perfusion parameters were calculated by averaging values of the 16 myocardial segments.

### Evaluation of reproducibility of LV myocardial perfusion parameters

Inter- and intra-observer variability for LV global and segments myocardial microcirculation perfusion measurements were determined in 60 random cases that included 43 T2DM patients and 17 control subjects. Intra-observer variability was obtained by comparison of perfusion parameters by the same observer with over 7 years of CMR experience in 2-month interval. To determine inter-observer variability, a second investigator with 7 years of CMR experience, who was unaware of the first observer’s results, compared independent measurements in the same population.

### Statistical analysis

All calculations were done with IBM SPSS Statistics for Windows version 24.0 (IBM Corporation, Armonk, NY, USA) and GraphPad Prism version 7.0a (GraphPad Software, San Diego, California, USA). Continuous variables were expressed as means with standard deviations or medians with inter-quartile ranges. One-way analysis of variance (One-way ANOVA) followed by Bonferroni’s post hoc-test or the Kruskal–Wallis rank test (when appropriate) were used to analyze differences among control subjects, T2DM(OCAD-) group, and T2DM(OCAD +) group, or among control subjects, T2DM(OCAD +) patients with Gensini score ≤ 64 group, and those patients with Gensini score > 64 group.

Univariable and multivariable linear regression analyses were used to investigate the independent and combined correlations of OCAD and T2DM with LV microvascular dysfunction, and evaluate independent predictors of impaired myocardial microcirculation perfusion among T2DM(OCAD +) patients. Candidate variables with *P* values of less than 0.1 in the univariable analysis were included in the final multivariable linear regression models with a stepwise selection procedure. Intra-class correlation coefficient (ICC) was used to assess intra- and inter-observer agreements. A two-tailed *P* value of < 0.05 was considered significant.

## Results

### Baseline characteristics of the study cohort

Table 1 summarizes baseline characteristics of the study cohort. T2DM(OCAD +) patients demonstrated longer disease duration [5 (0.75, 10) vs. 2 (0, 8) years, *P* < 0.05)], more likely to be older (61.42 ± 9.8 vs. 57.52 ± 12.34, *P* < 0.05), and more often men (80.7% vs. 58%, *P* < 0.05), as well as more smokers (50.5% vs. 35.1%, *P* < 0.05) than those T2DM(OCAD −) patients. Among T2DM(OCAD +) patients, 36(33%) patients had one-vessel disease, 29(26.6%) had two- vessel disease, and 44(40.4%) had three-vessel disease. In addition, the left ascending artery, left circumflex artery, and right coronary stenosis rate were 91.7%, 49.5%, and 64.2%, respectively. The median of Gensini score was 64 (37, 101) in those T2DM patients with OCAD.

**Table 1:** Baseline characteristics of the study cohort

	Control subjects	T2DM	T2DM	
	(n = 89)	(n = 297)	T2DM (OCAD** −**) (n = 188)	T2DM (OCAD** +**) (n = 109)
Male, n (%)	51 (57.3%)	197 (66.3%)	109 (58%)	88 (80.7%)^*§^
Age (years)	56.38 ± 8.63	58.95 ± 11.61	57.52 ± 12.34	61.42 ± 9.80^*§^
Systolic blood pressure (mmHg)	113.25 ± 11.17	132.51 ± 22.34^#^	134.26 ± 22.04^*^	129.50 ± 22.64^*^
Diastolic blood pressure (mmHg)	74.45 ± 6.29	81.27 ± 13.95^**#**^	82.11 ± 13.73^*^	79.83 ± 14.28^*^
Heart rate (beats/min)	72.45 ± 7.0	82.51 ± 15.31^**#**^	83.69 ± 15.87^*^	80.46 ± 14.14^*^
BSA (m^2^)	1.59 ± 0.16	1.73 ± 0.19^**#**^	1.70 ± 0.19^*^	1.77 ± 0.19^*^
BMI (kg/m^2^)	21.31 ± 3.31	25.39 ± 4.19^**#**^	25.28 ± 4.17^*^	25.58 ± 4.23^*^
Smoking, n (%)	10 (11.24%)	121 (40.7%) ^**#**^	66 (35.1%)^*^	55 (50.5%)^*§^
Diabetes duration (years)	−	3 (0, 9.75)	2 (0,8)	5(0.75, 10)^§^
TC (mmol/L)	3.79 ± 0.89	4.01 ± 1.37	4.13 ± 1.51	3.82 ± 1.06
TG (mmol/L)	1.14 ± 0.80	1.91 ± 1.51^**#**^	1.92 ± 1.51^*^	1.89 ± 1.53^*^
LDL (mmol/L)	2.16 ± 0.72	2.21 ± 1.03	2.25 ± 1.12	2.13 ± 0.85
HDL (mmol/L)	1.26 ± 0.30	1.12 ± 0.37^**#**^	1.16 ± 0.40	1.05 ± 0.30^*^
Fasting plasma glucose (mmol/L)	4.75 ± 0.63	9.12 ± 4.20^**#**^	8.53 ± 3.91^*^	10.14 ± 4.49^*§^
HbA_1c_, (%)	5.16 ± 0.30	7.43 ± 1.31^**#**^	7.06 ± 1.20^*^	8.08 ± 1.22^*§^
eGFR(ml/min/1.73m^2^)	90.08 ± 13.48	78.61 ± 22.38^**#**^	79.18 ± 23.51^*^	77.66 ± 20.39^*^
Gensini score	−	64 (37, 101)	−	64 (37, 101)
Location of coronary artery occlusion		100(91.7%)/54(49.5%)		100(91.7%)/54(49.5%)
(LAD/LCX/RCA)	−	/70(64.2%)	−	/70(64.2%)
Number of coronary arteries affected		36(33%)/29(26.6%)		36(33%)/29(26.6%)
(One/Two/Three-vessel)	−	/44(40.4%)	−	/44(40.4%)
OCAD treatment				
PCI	−	70 (64.2%)	−	70 (64.2%)
CABG	−	4 (3.7%)	−	4 (3.7%)
Statins, n (%)	−	95 (87.2%)	−	95 (87.2%)
Anti-thrombotic agents, n (%)	−	98 (89.9%)	−	98 (89.9%)
Calcium antagonists, n (%)	−	28(25.7%)	−	28(25.7%)
ACEI/ARB, n (%)	−	36 (33.0%)	−	36 (33.0%)
Beta blockers, n (%)	−	40 (36.7%)	−	40 (36.7%)
T2DM treatment				
Diet controlled, n (%)	−	86 (29%)	61 (32.4%)	25 (22.9%)
Insulin, n (%)	−	82 (27.6%)	47 (25%)	35 (32.1%)
GLP-1/DPP-4 inhibitor, n (%)	−	16 (5.4%)	11 (5.9%)	5 (4.6%)
a-Glucosidase inhibitor, n (%)	−	83 (27.9%)	49 (26.1%)	34 (31.2%)
Biguanides, n (%)	−	114 (38.4%)	63 (33.5%)	51 (46.8%)
Sulfonylureas, n (%)	−	52 (17.5%)	33 (17.6%)	19 (17.4%)

All values are presented as mean ± SD or n (%) or median (Q1-Q3)

*T2DM* type 2 diabetes mellitus, *OCAD* obstructive coronary artery disease, *BSA* body surface area, *BMI* body mass index, *HbA*_*1c*_ glycated hemoglobin, *TC* total cholesterol, *TG* triglycerides, *LDL* low-density lipoprotein, *HDL* high-density lipoprotein, *eGFR* estimated glomerular filtration rate, *LAD* left descending artery, *LCX* left circumflex artery, *RCA* right coronary artery; *PCI* percutaneous coronary intervention, *CABG* coronary artery bypass grafting; *ACEI* angiotensin converting enzyme inhibitor, *ARB* angiotensin receptor blocker; *GLP-1/DPP-4 inhibitor* glucagon-like peptide-1/dipeptidyl peptidase 4 inhibitor

^**#**^*P* < 0.05 T2DM patients vs. control subjects; *P < 0.05 vs. control subjects; ^§^*P* < 0.05 vs. T2DM(OCAD −) group

### CMR-derived parameters analysis in T2DM patients with and without OCAD and control subjects

CMR-derived parameters for observed groups are listed in Table 2. LVSVI and LVEF exhibited gradually decrease, while LVESVI showed a significant progressive increase from control subjects, through T2DM(OCAD −), to T2DM(OCAD +) patients (all *P* < 0.05). Compared with control subjects, T2DM patients both with and without OCAD had increased LVEDVI and LVMI (both *P* < 0.05).

**Table 2:** Differences of CMR-derived parameters among T2DM(OCAD −) patients, T2DM(OCAD +) patients, and control subjects

	Control subjects	T2DM	
	(n = 89)	T2DM (OCAD** −**) (n = 188)	T2DM (OCAD** +**) (n = 109)
LV geometry and function			
LVEF (%)	63.6 (58.2, 67.6)	54.5 (38.1, 63.3)^*^	36.8 (27.0, 53.8)^*§^
Indexed LV mass (g/m^2^)	42.4 (36.1, 49.0)	51.5 (42.8, 67.5)^*^	60.4 (48.1, 66.6)^*^
Indexed LVEDV (mL/m^2^)	79.7 (68.8, 87.7)	90.0 (74.1, 116.7)^*^	106.6 (78.4,140.7)^*^
Indexed LVESV (mL/m^2^)	28.2 (23.9, 33.4)	40.0 (27.3, 72.9)^*^	59.5 (35.8, 101.8)^*§^
Indexed LVSV (mL/m^2^)	49.9 (42.8, 54.4)	44.4 (32.8, 54.6)^*^	41.4 (32.0, 49.6)^*§^
First-pass perfusion parameters			
Basel			
Upslope	2.26 ± 0.91	1.85 ± 0.87^*^	1.52 ± 0.88^*§^
MaxSI	18.90 ± 5.86	18.55 ± 6.98	16.48 ± 6.62
TTM (s)	27.42 ± 10.39	33.86 ± 14.78^*^	39.08 ± 17.55^*§^
Mid-ventricular			
Upslope	2.69 ± 1.03	2.16 ± 0.93^*^	1.76 ± 0.96^*§^
MaxSI	23.01 ± 7.05	21.88 ± 7.28	19.91 ± 7.65^*^
TTM (s)	27.34 ± 10.52	33.72 ± 15.02^*^	40.14 ± 16.91^*§^
Apical			
Upslope	3.25 ± 1.23	2.69 ± 1.16^*^	2.26 ± 1.22^*§^
MaxSI	28.92 ± 8.41	27.42 ± 9.01	25.45 ± 9.44
TTM (s)	29.68 ± 10.82	35.12 ± 14.48^*^	41.88 ± 18.60^*§^
Global			
Upslope	2.66 ± 1.00	2.18 ± 0.92^*^	1.80 ± 0.96^*§^
MaxSI	22.80 ± 6.75	21.99 ± 7.18	19.97 ± 7.47^*^
TTM (s)	28.06 ± 10.31	34.21 ± 14.25^*^	40.18 ± 16.80^*§^

All values are presented as median (Q1-Q3) or mean ± SD. *LV* left ventricular, *EF* ejection fraction, *EDV* end-diastolic volume, *ESV* end-systolic volume, *SV* stroke-volume, *MaxSI* max signal intensity, *TTM* time to maximum signal intensity. *P < 0.05 vs. control group; ^§^*P* < 0.05 vs. T2DM (OCAD −) group

**Fig. 2:**
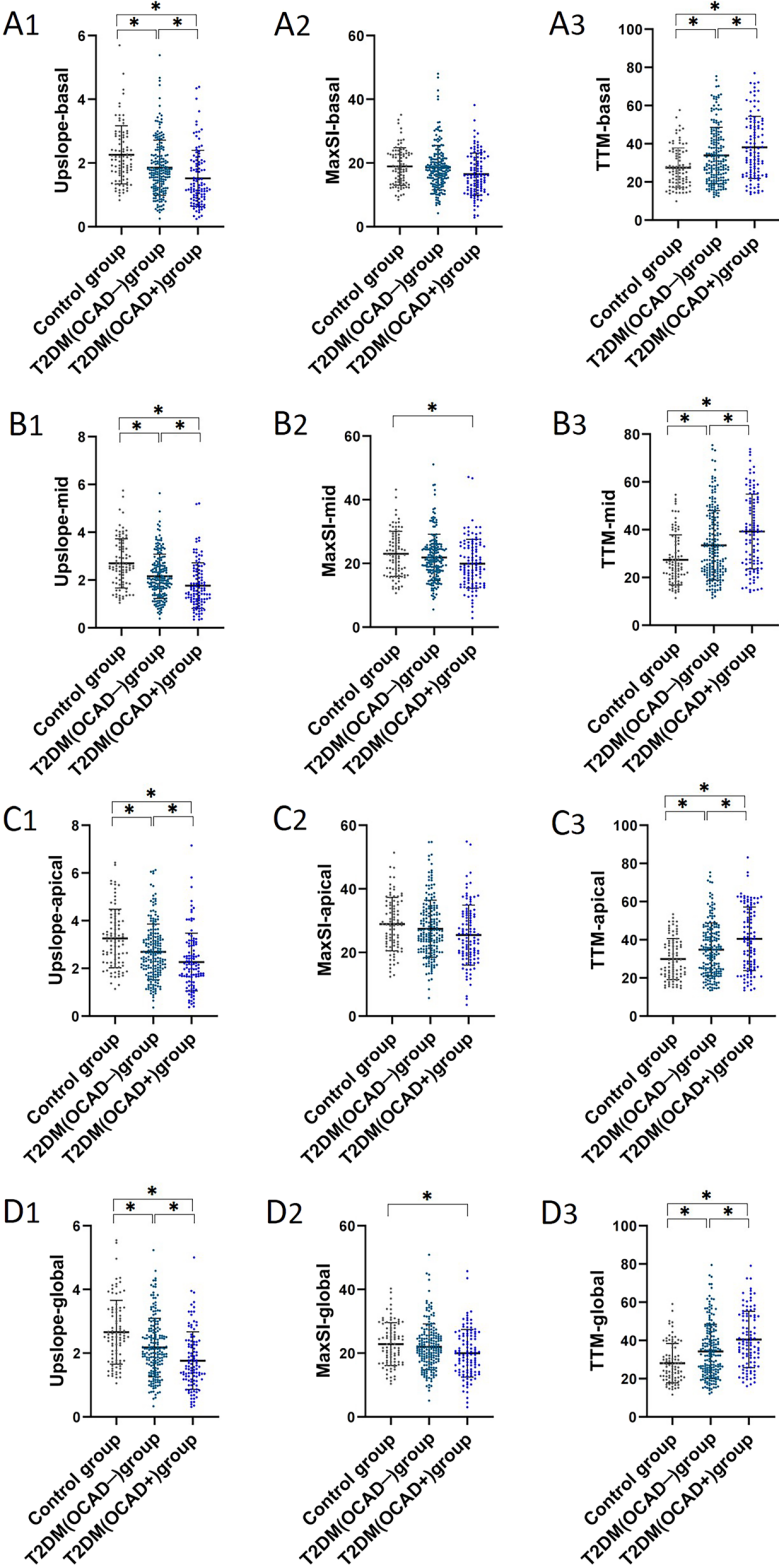
Comparison of first-pass perfusion parameters among control subjects, T2DM(OCAD −) patients, and T2DM(OCAD +) patients in the basal slice (A1-A3), mid-ventricular slice (B1-B3) and apical slice (C1-C3), as well as in global (D1-D3). Abbreviations as in Fig. 1. *P < 0.05

With regard to the LV myocardial first-pass perfusion, T2DM patients even without OCAD had worse microvascular function, which was supported by decreased upslope and increased TTM in global and all of three short-axis segments (basal, mid-ventricular, and apical) compared to the control subjects (all *P* < 0.05). Microcirculation dysfunction also existed and were exhibited a more marked impairment in the T2DM(OCAD +) patients than those T2DM(OCAD −) and control subjects, which was evident by a more reduced upslope and longer TTM in global and those above slices (all *P* < 0.05) (Figs. 1 and 2).

### First-pass CMR perfusion indices evaluation in T2DM(OCAD +) patients with different Gensini score group and control subjects

**Fig. 3:**
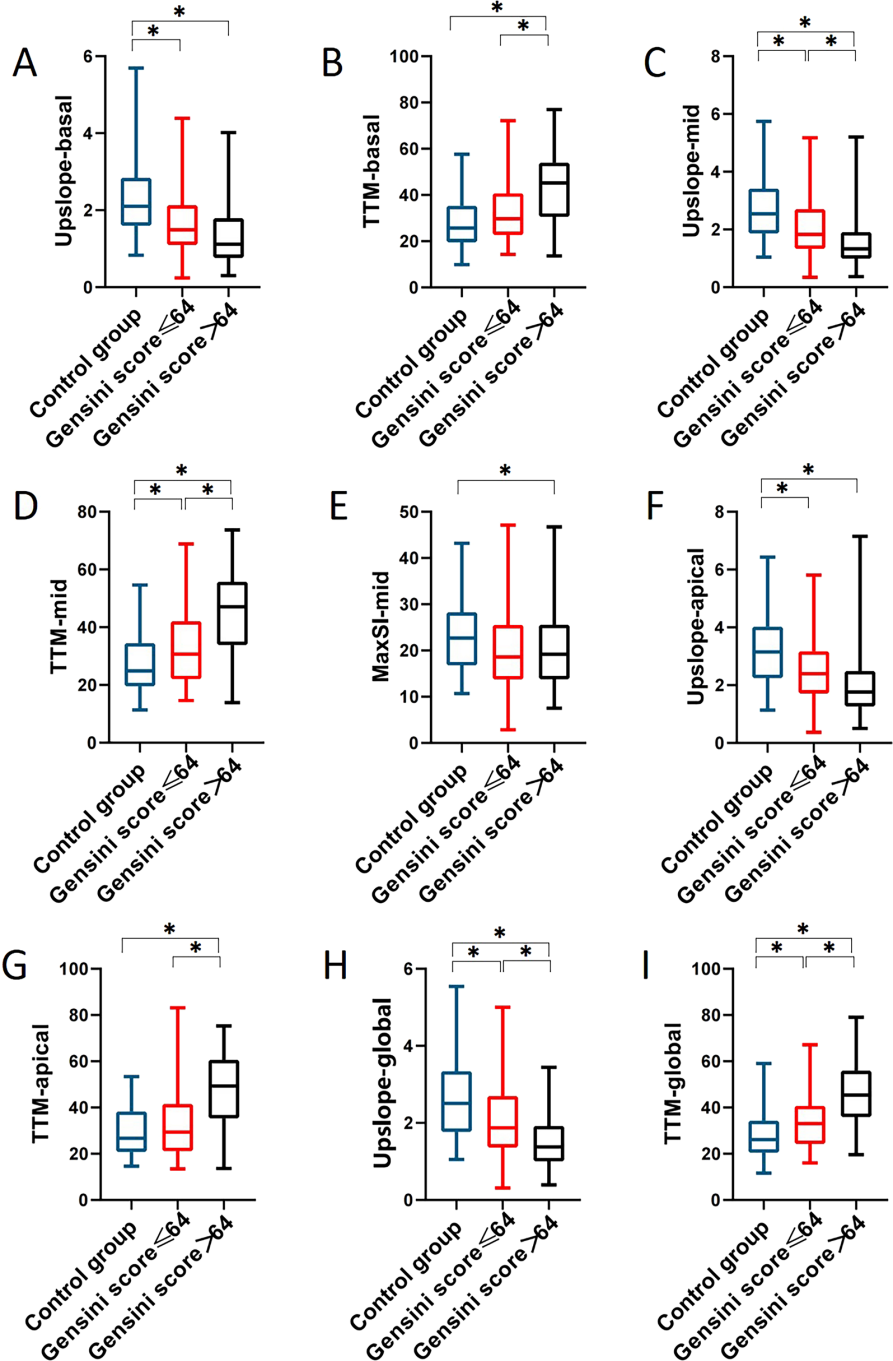
Differences of myocardial microvascular perfusion indices among control subjects, T2DM(OCAD +) patients with Gensini score ≤ 64, and those patients with Gensini score > 64. Comparison of upslope and TTM in the basal slice (A, B), apical slice (F, G) and in global (H, I), as well as upslope, TTM, and MaxSI in the mid-ventricular slice (C, D, E) among above-mentioned three groups. Abbreviations as in Fig. 1. *P < 0.05

T2DM (OCAD +) patients were categorized into two subgroups based on the median value (64) of the Gensini score: T2DM (OCAD +) patients with Gensini score ≤ 64 group (n = 53) and those with Gensini score > 64 group (n = 56). The LV global and segmental myocardial perfusion parameters for observed groups are exhibited in Fig. 3. In middle-ventricular segment, the upslope declined and the TTM prolonged progressively from control subjects, through T2DM (OCAD +) patients with Gensini score ≤ 64, to those with Gensini score > 64 (all *P* < 0.05). The same trends were observed in the global upslope and TTM among those above three groups (all *P* < 0.05). The upslope in basal and apical segments were similar between subgroups of T2DM (OCAD +) patients with different Gensini score (all *P* > 0.05).

### Incremental effect of OCAD on LV myocardial microvascular perfusion in T2DM patients

Multivariable linear regression analyses were performed to determine additive effect of OCAD on LV myocardial microvascular perfusion in T2DM patients using two models (Table 3). OCAD and T2DM were entered alone in model 1 with adjustment for sex, age, BMI, BSA, heart rate, systolic and diastolic blood pressure, smoking, and diabetes duration, demonstrating that the presence of OCAD or T2DM was independently associated with LV global upslope (OCAD: β =  − 0.15, *P* = 0.003, R^2^ = 0.19; T2DM: β =  − 0.23, *P* < 0.001, R^2^ = 0.22) and global TTM (OCAD: β = 0.14, *P* = 0.005, R^2^ = 0.23; T2DM: β = 0.19, *P* = 0.001, R^2^ = 0.23). With adjusted for those above demographic factors, OCAD and T2DM were entered together in model 2 showed that OCAD in the context of T2DM was significantly correlated with global upslope (R^2^ = 0.22) and global TTM (R^2^ = 0.24).

**Table 3:** Uni- and multi-variable linear regression analyses correlations of T2DM and OCAD with global coronary microvascular dysfunction

	Upslope-global					TTM-global (s)				
	Univariable		Multivariable			Univariable		Multivariable		
	r	*p*	β	*p-*value	*R2*	r	*p*	β	*p-*value	*R2*
Model 1: adjusted for demographic factors										
T2DM	− 0.26	< 0.001	− 0.23	< 0.001	0.22	0.25	< 0.001	0.19	0.001	0.23
OCAD	− 0.27	< 0.001	− 0.15	0.003	0.19	0.26	< 0.001	0.14	0.005	0.23
Model 2: adjusted for demographic factors										
T2DM	− 0.26	< 0.001	− 0.17	0.002	0.22	0.25	< 0.001	0.16	0.004	0.24
OCAD	− 0.27	< 0.001	− 0.10	0.037		0.26	< 0.001	0.11	0.037	

β is adjusted regression coefficient

Factors with *P* < 0.1 in the univariable analyses were included in the stepwise multiple liner regression model

*T2DM* type 2 diabetes mellitus, *OCAD* obstructive coronary artery disease, *TTM* time to maximum signal intensity

### Associations between global TTM and Gensini score, as well as clinical factors

**Fig. 4:**
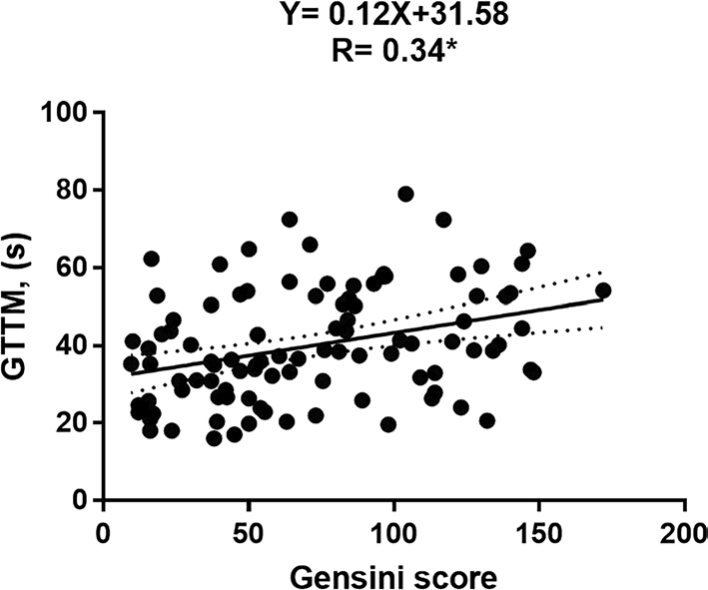
Association between global TTM and Gensini score in T2DM(OCAD +) patients. *T2DM* type 2 diabetes mellitus, *OCAD* obstructive coronary artery disease, *TTM* time to maximum signal intensity. *P < 0.001

As shown in Table 4, the univariable analysis in T2DM(OCAD +) patients exhibited that global TTM was positively associated with the Gensini score (r = 0.34, *P* < 0.001) (Fig. 4), and male sex (r = 0.398, *P* < 0.001), as well as smoking (r = 0.282, *P* = 0.003). In addition, there was a negative correlation between systolic blood pressure and global TTM (r =  − 0.35, *P* < 0.001). After controlling for confounding factors, including age, sex, BMI, BSA, heart rate, systolic and diastolic blood pressure, smoking, and diabetes duration, Gensini score remained the independent predictor of increased global TTM (β = 0.182, *P* = 0.042, R^2^ = 0.226).

**Table 4:** Associations between global TTM and Gensini score, as well as clinical factors in T2DM(OCAD +) patients

	TTM-global (s)				
	Univariable		Multivariable		
	r	*p*	β	*p-*value	*R2*
Gensini score	0.34	< 0.001	0.182	0.042	0.226
Sex	0.398	＜0.001	0.315	< 0.001	
Age	− 0.138	0.153	N/A	N/A	
BMI	− 0.097	0.317	N/A	N/A	
BSA	0.151	0.117	N/A	N/A	
SBP	− 0.353	< 0.001	− 0.226	0.013	
DBP	− 0.194	0.043	N/A	N/A	
Heart rate	− 0.034	0.729	N/A	N/A	
Smoking	0.282	0.003	N/A	N/A	
Diabetes duration	0.051	0.600	N/A	N/A	

β is adjusted regression coefficient

Factors with *P* < 0.1 in the univariable analyses were included in the stepwise multiple liner regression model

Abbreviations as in Tables 1 and 2

### Inter- and intra-observer variabilities of first-pass perfusion parameters

The inter- and intra-observer agreements of LV myocardial first-pass perfusion parameters were demonstrated in Table 5 and were considered excellent. The ICCs for inter-observer variability of LV myocardial perfusion in basal, mid-ventricular, apical segments, and global were 0.899 − 0.914, 0.909 − 0.951, 0.881 − 0.969, and 0.902–0.933, respectively. The ICCs for intra-observer variability of those above LV perfusion indices were 0.900 − 0.924, 0.910 − 0.968, 0.908 − 0.972, and 0.905–0.931, respectively.

**Table 5:** Intra-and inter-observer variabilities of first-pass perfusion parameters

	Intra-observer (n = 60)		Inter-observer (n = 60)	
	ICC	95% CI	ICC	95% CI
Basel				
Upslope	0.924	0.874 − 0.955	0.914	0.858 − 0.949
MaxSI	0.900	0.835 − 0.940	0.899	0.833 − 0.939
TTM (s)	0.913	0.857 − 0.948	0.908	0.848 − 0.945
Mid-ventricular				
Upslope	0.942	0.903 − 0.965	0.940	0.900 − 0.964
MaxSI	0.910	0.851 − 0.946	0.909	0.850 − 0.946
TTM (s)	0.968	0.946 − 0.981	0.951	0.917 − 0.971
Apical				
Upslope	0.936	0.893 − 0.962	0.924	0.869 − 0.956
MaxSI	0.972	0.952 − 0.983	0.969	0.948 − 0.982
TTM (s)	0.908	0.847 − 0.945	0.881	0.803 − 0.929
Global				
Upslope	0.905	0.844 − 0.943	0.904	0.842 − 0.942
MaxSI	0.931	0.885 − 0.959	0.933	0.888 − 0.960
TTM (s)	0.911	0.852 − 0.947	0.902	0.839 − 0.941

*ICC* intraclass correlation coefficient, *CI* confidence interval, *MaxSI* max signal intensity, *TTM* time to maximum signal intensity

## Discussion

The present study investigated impact of coronary obstruction on coronary microcirculation function in the context of T2DM assessed by first-pass perfusion CMR imaging. The following main findings were obtained: (1) T2DM was linked with impairment of myocardial microcirculation perfusion. (2) coronary obstruction, in the context of T2DM, might exacerbate myocardial microvascular dysfunction. (3) the presence of OCAD and the Gensini score were independent predictors of damaged microcirculation function.

### Effect of T2DM on microcirculation abnormalities

Multiple randomized clinical trials using different techniques have confirmed marked reduction of coronary flow reserve reflecting coronary microvascular function in diabetes patients, even in the absence of OCAD [8, 29, 30]. The present study using fist-pass perfusion CMR imaging supported the findings of those above investigations by demonstrating impaired LV myocardial microvascular perfusion with reduced upslope and prolonged TTM in global and all of three segments in T2DM(OCAD −) patients despite with preserved LVEF. In addition, our results were also in line with previous evidence that impaired microvascular function affected the left ventricle globally in addition to regionally in patients with T2DM [31].

Coronary microvascular abnormalities in T2DM involves multiple complex pathophysiological mechanisms including hyperglycemia, insulin resistance, and systemic inflammation, as well as autonomic dysfunction [29, 32, 33]. Furthermore, these abnormalities precede the onset of contractile dysfunction and clinically overt OCAD [34], and are also associated with poor cardiovascular prognosis [8]. Hence, early detection of coronary microvascular dysfunction in patients with T2DM might be helpful for early intervention to avoid adverse cardiovascular events [11].

### Incremental effect of coronary obstruction on microcirculation dysfunction in the context of T2DM

Several researches have founded patients with known coronary artery disease had impaired hyperemic flow and coronary flow reserve [9, 35, 36]. This present study extended these findings to patients with both OCAD and T2DM, a population that previously has rarely been explored, which exhibited that T2DM(OCAD +) patients had a more reduction in upslope and prolongation in TTM in global and all of three segments than those T2DM(OCAD −) patients and control subjects, suggesting that OCAD might exacerbate microcirculation damage in T2DM. Together, those above findings indicated that it is imperative to actively treat OCAD in patients with T2DM. Current therapies for diabetes patients with multi-vessel OCAD include percutaneous coronary intervention and coronary artery bypass graft (CABG), both of which have improved the prognosis among those patients [37, 38].

The pathophysiologic mechanisms of epicardial coronary artery obstruction on myocardial microcirculation dysfunction are poorly established, although several potential mechanisms have been proposed, including prearteriolar and arteriolar constriction and improper subepicardial prearteriolar dilatation in the existence of multiplied myocardial oxygen consumption [15, 16]. In addition, enhanced sympathetic activation is also likely to contribute to microvascular dysfunction under the coronary obstruction [16, 39]. Further researches in the context of T2DM are warranted on the underlying mechanisms of OCAD affecting coronary microcirculation function.

### First-pass perfusion CMR for evaluation of myocardial microvascular function

Currently, nuclear imaging and myocardial contrast echocardiography can be used to evaluate myocardial microcirculation function. However, both of these modalities are subject to certain limitations [15, 40]. First-pass perfusion CMR is a non-invasive and radiation-free modality for detecting myocardial ischemia with high diagnostic accuracy and spatial resolution [18–20, 40]. First-pass perfusion CMR parameters are derived from myocardial signal intensity-time curve including upslope, MaxSI, and TTM, which have been applied as semi-quantitative markers of tissue perfusion and associated with coronary microvascular function [18–20, 41].

In current study, there were excellent intra- and inter-observer agreements of the first-pass CMR perfusion technique for measuring upslope, MaxSI, and TTM among patients with T2DM. Previous studies have indicated that coronary microcirculation function, as evaluated by first-pass CMR myocardial perfusion, might serve as an additional marker for prognosis evaluation and therapeutic response [18, 20, 42]. As coronary microvascular dysfunction is an independent and strong risk factor of clinical deterioration and death in patients with T2DM and OCAD [9], future studies will focus on using this technique to evaluate the prognosis among those patients.

### Independent predictors of microvascular dysfunction

Much less is known about the interrelationship between the coronary microcirculation and the epicardial coronary arteries [12–14]. In our data, the presence of OCAD was an independent predictor of myocardial microcirculation dysfunction in the context of T2DM. A better comprehension of the relationship between the epicardial coronary artery obstruction and microvascular disorder would be beneficial to effectively diagnose and treat microvascular dysfunction in patients with T2DM. Current researches show that multi-lineage cell therapy is a novel, translatable approach and is expected to improve microvascular disease in diabetic patients [43, 44].

Our results suggested that T2DM(OCAD +) patients with Gensini score > 64 might had a more severe microvascular dysfunction than those patients with Gensini score ≤ 64 and control subjects. Increasing evidence has demonstrated that the extent and severity of coronary artery stenosis are associated with decreased myocardial microcirculation perfusion among patients with known or suspected coronary artery disease [45–47].

Data from experimental study in pig model conducted by Fearon et al. [48] have found that microvascular resistance is not affected by the epicardial coronary artery stenosis severity. Conversely, by analyzing the correlation between the Gensini score and microcirculation function, the current study demonstrated that global TTM prolonged progressively along with the increase of the Gensini score which is widely used for quantifying the severity of OCAD [27]. Possible reason for this discrepancy might be explained by the differences between animal models and humans. Taken together, our observations implied that the severity of the OCAD was also a predictor of coronary microcirculation dysfunction. Consequently, early prevention and active intervention of OCAD are expected to improve coronary microvascular function [15, 16] in patients with T2DM (OCAD +).

## Limitations

Our study has several limitations. Firstly, this was a mono-centric study, which carries inherent bias in terms of selection. A multicenter study is desirable to generalize our observations. Secondly, as a cross-sectional analysis, we cannot know the evolution of cardiac microcirculation damage in patients with T2DM(OCAD +) overtime with the progression of OCAD. Further follow-up studies will be performed to address this question. Thirdly, stress CMR perfusion imaging has been reported to prospectively evaluate myocardial ischemia in some cardiovascular diseases [19, 20, 42]. Due to the inherent limitations of retrospective design, future prospective trials will use this imaging modality to explore the incremental effect of OCAD on myocardial microcirculation function among T2DM patients. Finally, not all of our T2DM(OCAD −) patients were performed ICA examination or coronary computed tomography angiography to exclude OCAD, and control individuals were not performed those above examination to exclude no clinical symptoms coronary artery obstruction. According to comprehensive evaluation of the patients by clinical history, laboratory examination, electrocardiography, and echocardiography, OCAD was deemed to be unlikely in those patients [21].

## Conclusions

Coronary artery obstruction in the context of T2DM might exacerbate myocardial microcirculation damage. The presence of OCAD and Gensini score were independent predictors of myocardial microvascular dysfunction. Early detection myocardial microcirculation abnormalities using first-pass perfusion CMR in T2DM patients, especially when comorbid with OCAD, would be essential for timely therapeutic interventions.

## Data Availability

The datasets used and analyzed during the current study are available from the corresponding author on reasonable request.

## Abbreviations

T2DM: Type 2 diabetes mellitus
OCAD: Obstructive coronary artery disease
CMR: Cardiac magnetic resonance
MaxSI: Max signal intensity
TTM: Time to maximum signal intensity
CMD: Coronary microvascular dysfunction
PG: Plasma glucose
LV: Left ventricular
ICA: Invasive coronary angiography
bSSFP: Balanced steady-state free precession
TR: Repetition time
TE: Echo time
FOV: Field of view
LVEDV: LV end-diastolic volume
LVESV: LV end-systolic volume
LVSV: LV stroke volume
LVEF: LV ejection fraction
BSA: Body surface area
ICC: Intra-class correlation coefficient
